# Facilitating Discovery: The Role of Society Journals in Collaborative Science

**DOI:** 10.1534/g3.111.001859

**Published:** 2012-02-01

**Authors:** Lauren M. McIntyre, Dirk-Jan de Koning

**Affiliations:** * University of Florida, Gainesville, Florida 32610, and; † Swedish University of Agricultural Sciences, SE-750 07 Uppsala, Sweden

Scientific progress requires hypotheses and ideas, but it is enabled by resources. Genetic resource panels (GRPs) like the “Collaborative Cross” are a unique resource for the scientific community. Because this resource allows repeated generation of particular genetic combinations, the impact of DNA sequence variation on a wide range of phenotypes can be readily studied. Renewable resource populations have been created for flies, maize, Arabidopsis, yeast, worms, and mice (*e.g.,* Giaever *et al*. 2002; Kamath *et al*. 2003; Complex Trait Consortium 2004; Macdonald and Long 2007; Ayroles *et al.* 2009; Kover *et al.* 2009; McMullen *et al.* 2009). With them phenotypic data for transcription, metabolites, proteins and whole organism can be linked to underlying genetic polymorphisms. These resources give scientists insight into how genetic variation percolates through the biological layers, from cells to the whole organism, from molecules to networks. One critical requirement for realizing the potential of these accumulating resources is that all data be publicly available, preferably accompanied with a set of online tools for mining them. The February 2012 issues of Genetics and G3: Genes | Genomes | Genetics highlight the launch of the Collaborative Cross for mice and present the first exciting results, both data- and methods-driven, issuing from this cross. The most notable predecessor of the Collaborative Cross is the BXD panel of recombinant inbred lines for which a mass of phenotypic, transcriptomic and genetic data are publically available online. Emerging technologies that allow measurements on single cells to complex behaviors mean that geneticists have the opportunity not only to revel in biological complexity but to deconstruct and understand it.

How can society-sponsored, peer-edited journals like ours help the community in making the most of these exciting resources? GENETICS and G3 are sister journals committed to the highest level of scientific rigor. Both journals are committed to open access of all data used in their articles, and require that all the data are submitted to a public repository, or available as a supplement, thereby enabling others to connect the dots in their own experiments and analysis. We pay more than just lip service to our data policy. We believe it serves scientists and science well.

Data sharing is particularly crucial for GRPs like the Collaborative Cross. If some of the pieces of the puzzle have been taken off the board and are only available only through a complex compact, then the puzzle is unlikely to be completed, and the community resource is compromised. Peer editors are invested in ensuring that these resources live up to their potential.

The 15 articles on GRPs for the mouse in this month's issues of GENETICS and G3 illustrate The Editors’ vision for one way in which society-sponsored peer editing adds value for the scientific community. All the data associated with these papers is publicly available, and the authors are committed to its widest possible distribution. We are proud to provide a venue for the publication of such important work, and we will continue to facilitate publication of high-quality studies focused on this unique resource. The Editors of GENETICS and G3 are working together to maximize the full impact of such large scale endeavors. The scientific community needs new tools for analysis, as well as creative experiments that unlock the promise of GRPs. We welcome submissions of manuscripts that answer these broad challenges. We also invite submission of manuscripts that describe the use of the resources presented in this month's issues of Genetics and G3.

The Editors of GENETICS and G3 are committed to publishing high-quality science and to supporting the scientific community. To that end, we seek to collaborate with scientific communities engaged in projects to develop genetic resources. We are developing ways to tag papers using specific metadata identifiers so all papers involving a particular GRP can be quickly located and collated. Look for our tags for mouse-GRP, mouse-CC, and mouse-DO. Tell us more about ways you think the journals of the Genetics Society of America can collaborate with and support our community.
